# The aqueous extract of *Phellinus igniarius* (SH) ameliorates dextran sodium sulfate-induced colitis in C57BL/6 mice

**DOI:** 10.1371/journal.pone.0205007

**Published:** 2018-10-05

**Authors:** Yuqing Sun, Shi Zhong, Jiaqi Yu, Jianxun Zhu, Dongfeng Ji, Guiyan Hu, Chongming Wu, Yougui Li

**Affiliations:** 1 Sericultural Research Institute, Zhejiang Academy of Agricultural Science, Hangzhou, China; 2 Pharmacology and Toxicology Research Center, Institute of Medicinal Plant Development, Chinese Academy of Medical Sciences & Peking Union Medical College, Beijing, China; University of South Carolina School of Medicine, UNITED STATES

## Abstract

*Phellinus igniarius*, which is called Sanghuang in Chinese, is a fungal herb widely used in Traditional Chinese Medicine to treat stomachache, inflammation and tumors. Recent studies have demonstrated the antitumor, anti-diabetic, anti-inflammatory and immunity-modulating activities of *P*. *igniarius*. In the present study, we investigated that ameliorating effect of the aqueous extract of *P*. *igniarius* fruiting body (SH) on dextran sodium sulfate (DSS)-induced colitis in C57BL/6 mice. Treatment with SH (250 and 400 mg/kg) for 8 weeks effectively alleviated the pathological indicators of colitis such as bodyweight reduction, disease activity index score, shortening of colon length and abnormal colon histology. The plasma levels of lipopolysaccharide (LPS) and inflammatory factors such as interleukin-6 (IL-6), IL-1β and tumor necrosis factor (TNF)-α were all significantly reduced. Supplementation of SH (10 mg/L) also inhibited LPS-elicited IL-1β production by RAW264.7 macrophages. Real-time PCR and western blot showed that treatment with SH significantly inhibited the phosphorylation of nuclear factor kappa B inhibitor alpha (IκBα) and decreased the expression of IL-6/IL-1β-maturation genes such as apoptosis-associated speck-like protein (ASC3) and caspase-1 in the colon of DSS-induced colitis mice. These results suggest that SH is adequate for the treatment of colitis. Inhibiting the expression and release of inflammatory factors may participate in the colitis-ameliorating effect of SH.

## Introduction

Inflammatory bowel disease (IBD) is a chronic remittent and relapsing inflammatory condition, which is characterized by chronic inflammation in the colon and mainly comprises ulcerative colitis (UC) and Crohn’s disease (CD)[1]. The typical symptoms of IBD are diarrhea, rectal bleeding, intermittent abdominal pain and weight loss, seriously deteriorate the quality of life and lead to increased risk of colon cancer[1]. The precise pathogenesis of IBD has not been completely elucidated, but the chronic relapsing inflammation is implicated to the exaggerated immune response to gut microbiota. This dysregulated response results in increased expression and secretion of pro-inflammatory cytokines, such as tumor necrosis factor-α (TNF-α), interferon-γ, interleukin (IL)-1β and IL-6, which ultimately damages colon tissues[2].

Lipopolysaccharide (LPS) is an important immune stimulatory factor that is derived from bacteria and induces systemic inflammation[3]. Intensive investigations have shown that LPS triggers inflammation through a series of interactions with multiple proteins including Toll-like receptor 4 (TLR4), Myeloid differentiation primary response 88 (MyD88), TNF receptor associated factor 6 (TRAF6), mitogen-activated protein kinases (MAPKs) and nuclear factor kappa B (NFκB), which ultimately results in enhanced expression of pro-inflammatory cytokines such as IL-1β and IL-6 [4, 5]. LPS also stimulates inflammatory through activation of the NACHT, LRR and PYD domains-containing protein 3 (NLRP3) inflammasome which consists of NLRP3, caspase-1, and apoptosis-associated speck-like protein (ASC3) [6].

The medicinal mushroom *Phellinus igniarius* (Linnearus: Fries) Quél, which is called Sanghuang in Chinese, is a basidiomycetous fungus belonging to the Hymenochaetaceae family in Basidiomycetes [7, 8]. The fruiting body of *P*. *igniarius* has been widely used in Traditional Chinese Medicine to treat stomachache, inflammation and tumors [8]. Multiple investigations have demonstrated that the extract of *P*. *igniarius* possesses antitumor, anti-diabetes and immunity-modulating effect [7–14]. Polyphenols and polysaccharides are implicated as the major active components of *P*. *igniarius* [9, 11]. Although multiple pharmacological activities of *P*. *igniarius* have been demonstrated [12], whether it is beneficial for the treatment of colitis is largely unknown.

In the present study, the effect of the aqueous extract of *P*. *igniarius* fruiting body (SH) on dextran sodium sulfate (DSS)-induced colitis was investigated in C57BL/6 mice. The therapeutic efficiency was evaluated by monitoring the colitis symptoms, histopathological damage of colon and plasma levels of inflammatory cytokines. The potential mechanisms underlying SH-elicited anti-inflammation effects were also explored.

## Materials and methods

### Preparation of *Phellinus igniarius* extract (SH)

The fruiting body of cultured *P*. *igniarius* was obtained from Xin Hong Biotech Corp., Ltd. (Hai Ning, Zhejiang province, China). The fruiting body was cut into slices and dried. Then the samples were boiled with water (w/v = 1:10) for 1 h, the filtrate was separated with filter, and the residue was further extracted under the same condition twice. The filtrates collected from three separate extractions were combined, concentrated rotary evaporation and lyophilized by freeze vacuum drying. The dried extract of *P*. *igniarius* was collected, weighed and stored at -20°C until use. One kilogram of *P*. *igniarius* fruiting body yielded 226 g of *P*. *igniarius* aqueous extract (SH). The polyphenol content in SH was 37.23% as determined by colorimetric method[15].

### Animals and experiment design

All the animal experiments were performed in accordance with the National Institutes of Health regulations for the care and use of animals in research and were approved by the Medical Ethics Committee of Zhejiang Academy of Agricultural Sciences (No.ZAAS-20171005021). All efforts were taken to minimize animals suffering.

Forty male C57BL/6 mice (6-week-old, male) were purchased from Shanghai Experimental Animal Center (Shanghai, China). The mice were acclimatized under standard conditions for 1 week, fed with standard laboratory chow and kept in an animal room with 12-h light/12-h dark cycles. Animals were then randomly divided into 4 groups (n = 10 per group), which are named Normal, DSS, DSS+SH-250 and DSS+SH-400. Chronic colitis was established by supplementation of dextran sodium sulfate (DSS) (molecular weight 36–50 kDa, MP Biologicals, USA) into drinking water (1.0% w/v) for 48 days. The Normal group was not induced colitis and was administered with drinking water. The DSS group was established colitis and continued to drink DSS only. The DSS+SH-250 and DSS+SH-400 groups were established colitis and treated with SH at dosage of 250 and 400 mg/kg/day, respectively. Body weight and disease activity index were assessed every 3 days. At the end of the experiment, animals were fasted overnight and blood samples were collected for estimation of plasma parameters by kits. Animals were then euthanized by spinal dislocation after ether anesthesia, and the colon tissue was taken for histological and biochemical analysis.

### Evaluation of disease activity index (DAI)

The DAI was used for evaluating the grade and extent of intestinal inflammation [16]. Stool consistency and bloody stool were monitored every 3 days for determination of DAI. Each parameter was scored as follows: diarrhea (0, normal; 2, loose stools; 4, watery diarrhea) and bloody stool (0, normal; 2, slight bleeding; 4, gross bleeding). The DAI score ranged from 0 to 8 (total score).

### Histological analysis

The entire colon of each mouse was taken, and its length was immediately recorded. Then a piece of colon tissue was removed from each mouse, fixed in 4% paraformaldehyde in PBS, embedded in paraffin, and cut into 5-μm-thick sections. The sections were stained with hematoxylin and eosin (H&E) and the histology of colon tissue was examined under a light microscopy (Nikon Co., Japan).

### Cell experiment

RAW264.7 cells were originated from the American Type Culture Collection (ATCC) (Manassas, VA, USA) and obtained from the Peking Union Medical College (Beijing, China). RAW264.7 cells, were maintained in DMEM containing 10% FBS at 37 °C and 5% CO_2_. When grown to 70–80% confluence, cells were kept in serum-free DMEM and pre-incubated with SH (10 mg/L) for 24 h, then treated with LPS (10 ng/mL) for 4 h. The blank group was incubated with serum-free DMEM alone. After LPS treatment, the medium level of IL-1β was determined using ELISA kit (Cell signaling, Beverly, USA) according to the instruction of manufacturer.

### Measurement of plasma cytokines

The plasma concentrations of lipopolysaccharide (LPS), interleukin-6 (IL-6), IL-1β and tumor necrosis factor (TNF)-α were assessed using commercially available enzyme-linked immunosorbent assay (ELISA) kits (R&D Systems, Minneapolis MN) according to the manufacturer’s instructions. Biotin-conjugated secondary antibodies were used, followed by streptavidin-HRP (Amersham Biosciences, Arlington Heights, IL) and an OPD substrate system (Sigma). The colorimetric reaction was read in an automated ELISA microplate reader (Versamax, Molecular Devices) at 492 nm.

### Real-time PCR

The colon tissues were homogenized using a tissue homogenizer. The total RNA extraction, cDNA synthesis and real-time PCR were performed as previously described [17]. Briefly, the total RNA was isolated and the cDNA was synthesized from the colon tissue, and these were processed using a universal mRNA Purification Kit (TaKaRa, Japan) and RT reagent Kit with gDNA Eraser (TaKaRa, Japan), respectively. Real-time PCR was performed using the SYBR Fast qPCR Mix (TaKaRa, Japan) via the StepOne Plus Real-time PCR system (Life Technologies, Carlsbad, CA, USA). The PCR volume was 15 μL, and it included 0.3 mL of each primer (10 nmol/L), 7.5 μL of the SYBR qPCR mix, 0.3 μL of ROX reference dye, 2.0 μL of the cDNA template, and 4.6 μL of easy dilution buffer (TaKaRa, Japan). The PCR program included 30 s at 95°C, then 40 cycles of 95°C for 5 s, and finally 62°C for 34 s. At least three independent biological replicates were performed to check the reproducibility of the data. The gene-specific primers used for quantitative PCR are listed in Table 1.

**Table 1:** Primers used in quantitative real-time reverse transcription-PCR.

Primer	Sequence 5’-3’	PCR product size(bp)
ASC-F	TTCTGTGACCCTGGCAATGAG	198
ASC-R	CTGGAGTCGTATGGCTTGGAG	
Caspase-1-F	CAGGCAAGCCAAATCTTTATCACT	173
Caspase-1-R	GTGCCATCTTCTTTGTTCTGTTCTT	
GAPDH- F	CAGCCTTCCTTCTTGGGTAT	105
GAPDH- R	CTGTGTTGGCATAGAGGTCTT	
IKKα- F	CACAGCCTCTAACTCCATCTA	114
IKKα- R	AGCGTGAAACAGGAATAAATA	
IKKβ- F	ACTGGAAGGCTGGGACATTAG	107
IKKβ- R	TGAAGATCGCCTGTAGCAAAG	
IL-1β-F	TGTGTTTTCCTCCTTGCCTCTGAT	105
IL-1β-R	TGCTGCCTAATGTCCCCTTGAAT	
IL-6-F	GAGGATACCACTCCCAACAGACC	141
IL-6-R	AAGTGCATCATCGTTGTTCATACA	
IRAK-2- F	CCGGGGTATTGGAAGATGAGC	240
IRAK-2- R	TGGGGAAGGCAGTGGTGAAAG	
JNK1- F	TCCTCCAAATCCATTACCTCC	149
JNK1- R	CTCCAGCACCCATACATCAAC	
JNK2-F	AGGACGAGTTCACGGTAGGCT	190
JNK2-R	GCCATCTTTGATAACACCACT	
MEKK-1- F	CTCCAAAGTCTGCAATTCTCA	153
MEKK-1- R	CTTTCAAGGAGTCAGTCGTCA	
MyD88-F	GTAGAGGCAGGAGAATCAGGAG	161
MyD88-R	ACAGGTAGAGAAAAGAGAGGAGAAA	
NLRP3-F	GTCTGGAAGAACAGGCAACAT	144
NLRP3-R	AGAACTGTCATAGGGTCAAAACG	
p38α-F	GTTTCAGTCCATCATTCACGC	93
p38α-R	ACATCCAACAGACCAATCACAT	
p65-F	GTGTCTTGGTGGTATCTGTGC	166
p65-R	GATCTGTTTCCCCTCATCTTT	
TAK1- F	CTGGATGATCGGGACTAAAGA	129
TAK1- R	GGATAAAGCAAGTGATGGAGC	
TLR-4-F	GGAACAAACAGCCTGAGACAC	151
TLR-4-R	CAAGGGATAAGAACGCTGAGAA	
TRAF6- F	CCAGGGGAGGTGGCTGTCATA	209
TRAF6- R	GGAAGATTGGCAACTTTGGGATG	
Ubc13-F	TGCTGGGGACCACTTATCTTT	205
Ubc13-R	CAACGCCCGTTATTTTCATGT	
UEV1A-F	TTCGGTTTTCATAGATTGTTCGTG	111
UEV1A-R	GAGTAGGCGACGGCACAGTTA	
ZO-1-F	AGGACACCAAAGCATGTGAG	192
ZO-1-R	GGCATTCCTGCTGGTTACA	

### Western blot

Western blot analysis was performed on tissue extract as previously reported[18]. Briefly, tissue samples were lysed in lysis buffer containing 10% glycerol, 1% Triton X-100, 135 mM NaCl, 20 mM Tris (pH 8.0), 2.7 mM KCl, 1 mM MgCl_2_, and protease and phosphatase inhibitors (0.5 mM PMSF, 2 μM pepstatin and 2 μM okadaic acid). Aliquots of samples were subjected to SDS-PAGE followed by transfer to polyvinylidene difluoride (PVDF) membranes (Amersham Pharmacia, Uppsala, Sweden). Immunoblotting was performed with respective antibodies (1:1000). Following incubation with horseradish peroxidase-conjugated secondary antibody (SigmaAldrich, Shanghai, China), proteins were detected with ECL plus kits (Amersham, Piscataway, NJ, USA).

### Statistical analyses

Data were presented as mean ± standard error (sem). Statistical analyses were performed using Student's *t*-test using SPSS, version 16 (SPSS Inc., Chicago, IL, USA). A P-value < 0.05 was considered statistically significant.

## Results

### SH attenuated the symptoms of DSS-induced chronic colitis

**Fig 1:**
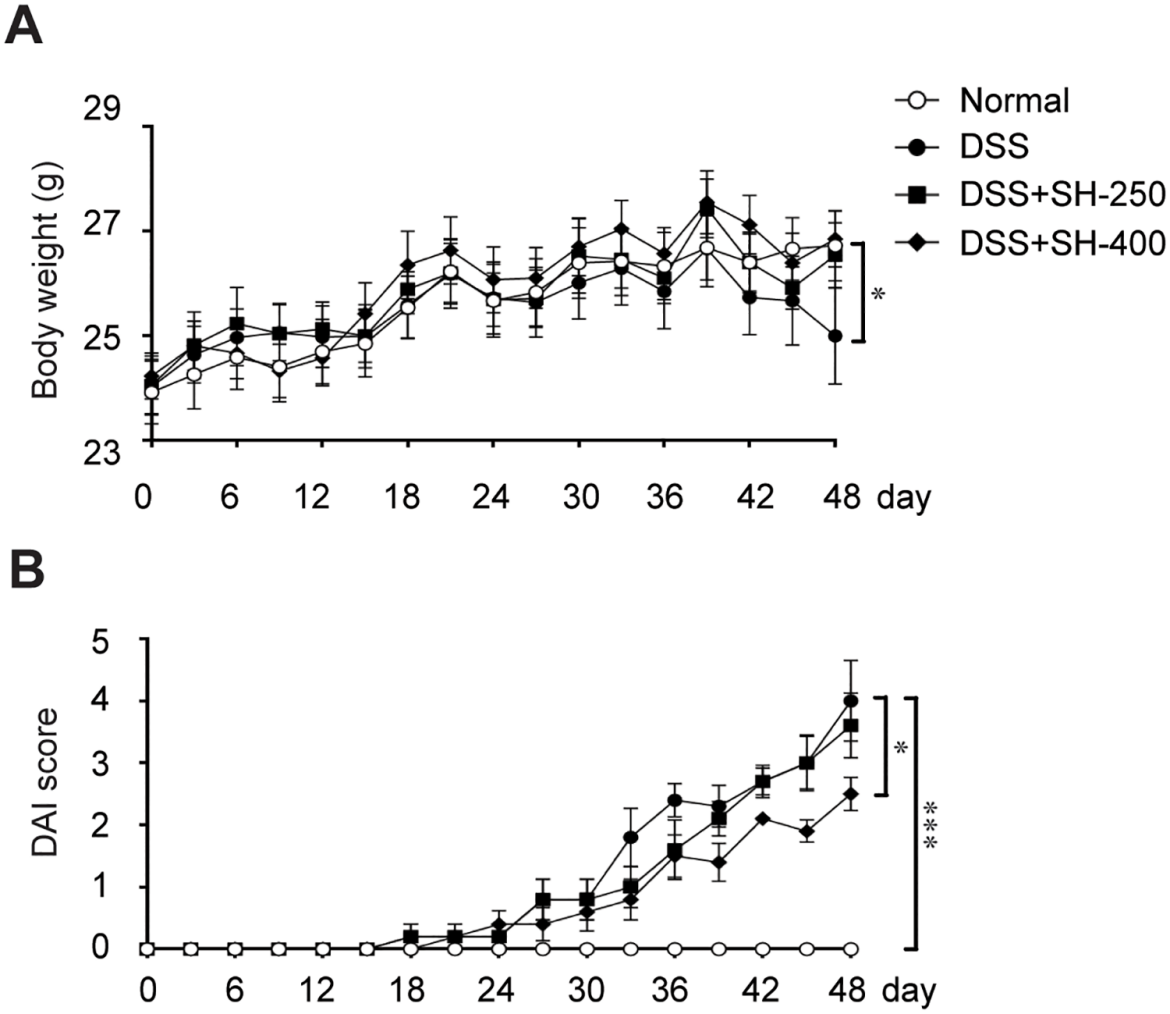
SH attenuated the symptoms of DSS-induced chronic colitis. (A) Changes of body weight. (B) Disease activity index (DAI) score. Data are shown as means ± sem. *P<0.05, ***P<0.001.

The clinical symptoms of colitis were estimated by DAI score and body weight reduction. The body weight of all animals increased constantly in the first 39 days. Afterward, the body weight of DSS group declined sharply and was significant lower than the Normal group. Treatment with SH (400 mg/kg) significantly prevented the reduction of body weight (Fig 1A). Similarly, the DAI score of DSS group increased after DSS-drinking for 18 days, and the DAI score reached 4 after induction for 8 weeks (Fig 1B), revealing severe diarrhea and gross anal bleeding. Treatment with SH decreased the DAI score in a dose-dependent manner. The DAI score of SH-400 group was significantly lower than that treated with DSS alone (Fig 1B). These results suggested that SH was effective to ameliorate clinical symptoms of colitis.

### SH improved the histopathological damage of colon

**Fig 2:**
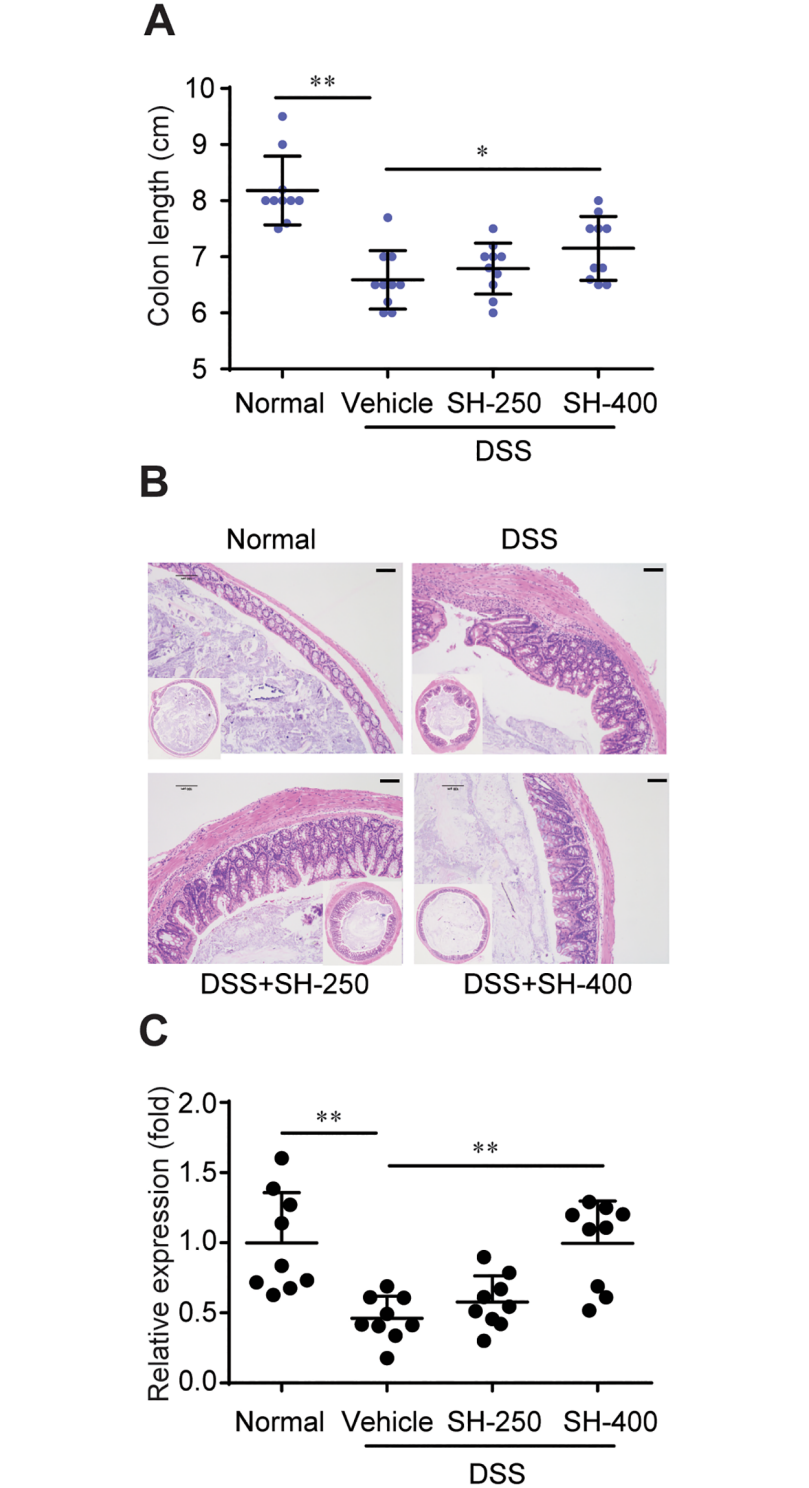
SH improved the histopathological damage of colon. (A) Colon length. (B) H&E staining of colon tissue sections. (C) Relative mRNA levels of tight junction protein ZO-1 in colon tissue. Data are shown as means ± sem. *P<0.05, **P<0.01.

Shortening of the colon length reflects the extent of colon damage in DSS-induced colitis [19]. The average colon length was approximately 8.2 cm in healthy controls, while it was reduced to 6.6 cm in mice with DSS-induced colitis. Treatment with SH (400 mg/kg) significantly increased the colon length as compared to DSS group (Fig 2A).

Histological analysis also confirmed the alleviating effect of SH on colon pathology. The colon of Normal group revealed large numbers of goblet cells, preserved crypts and muscularis propria, and intact mucosal lining. Supplementation of DSS led to significant colonic damage, as evidenced by loss of epithelial barrier, decrease in the number of crypts and goblet cells, and submucosal edema (Fig 2B). Treatment with SH improved these pathological changes of colon such as abnormal crypts, loss of epithelial cells and goblet cells, and inflammatory infiltration (Fig 2B).

The intestinal tight junctions are key structures defending intestine against leaking of adverse macromolecules such as LPS. We quantified the relative expression of tight junction protein ZO-1 in colon tissue by realtime PCR. The results showed that the expression of ZO-1 in DSS group was significantly lower than normal mice, and treatment with SH (400 mg/kg) significantly increased the expression of ZO-1 (Fig 2C). This indicates that SH has protective effect on intestinal mucosal barrier.

### SH reduced the levels of inflammatory cytokines in mice with DSS-induced colitis

**Fig 3:**
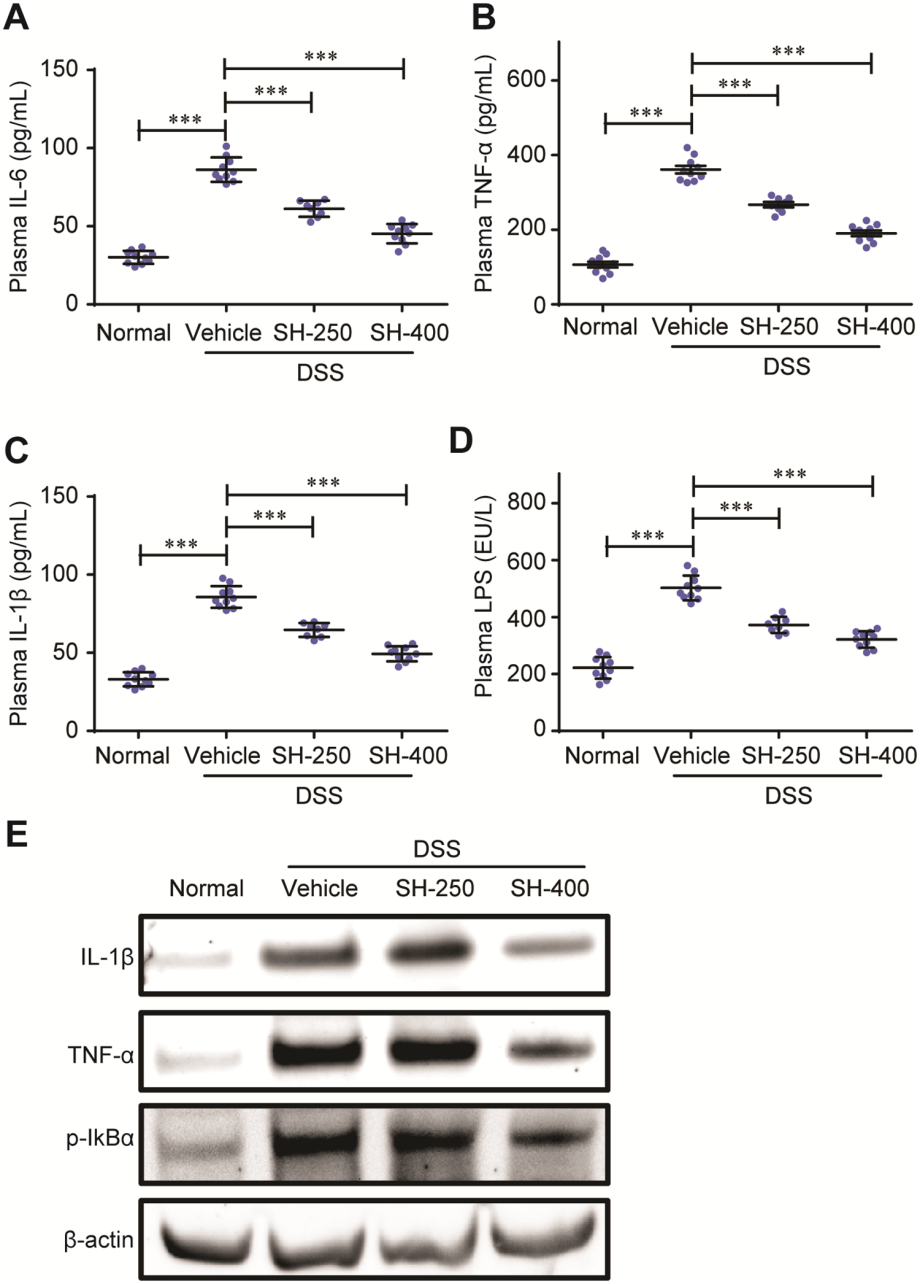
SH reduced the levels of inflammatory cytokines. The plasma levels of IL-6 (A), TNFα (B), IL-1β (C) and LPS (D) were determined by ELISA. (E) Western blot for colon levels of IL-1β, TNFα and phosphorylated IκBα (p-IκBα). Data are shown as means ± sem. ***P<0.001.

We first quantified the plasma levels of inflammatory cytokines such as IL-6, IL-1β, TNF-α, and LPS by enzyme-linked immunosorbent assay (ELISA). Supplementation with DSS remarkably increased the plasma levels of IL-6, IL-1β and TNF-α, revealing a severe inflammatory condition. DSS also increased the plasma concentration of LPS, a potent inflammation inductor that was derived from gram-negative gut bacterium (Fig 3A–3D). Treatment with SH significantly and dose-dependently decreased the plasma levels of IL-6, IL-1β and TNF-α, exhibiting potent anti-inflammatory activities. The administration of SH also significantly lowered the LPS level as compared to the vehicle control (DSS) group. We also determined the protein levels of IL-1β and TNF-α in colon tissues by western blot analysis. The results showed that treatment with SH (400 mg/kg) significantly decreased the contents of IL-1β and TNF-α in colon (Fig 3E).

### SH inhibited LPS-induced IL-1β production by RAW264.7 macrophages

**Fig 4:**
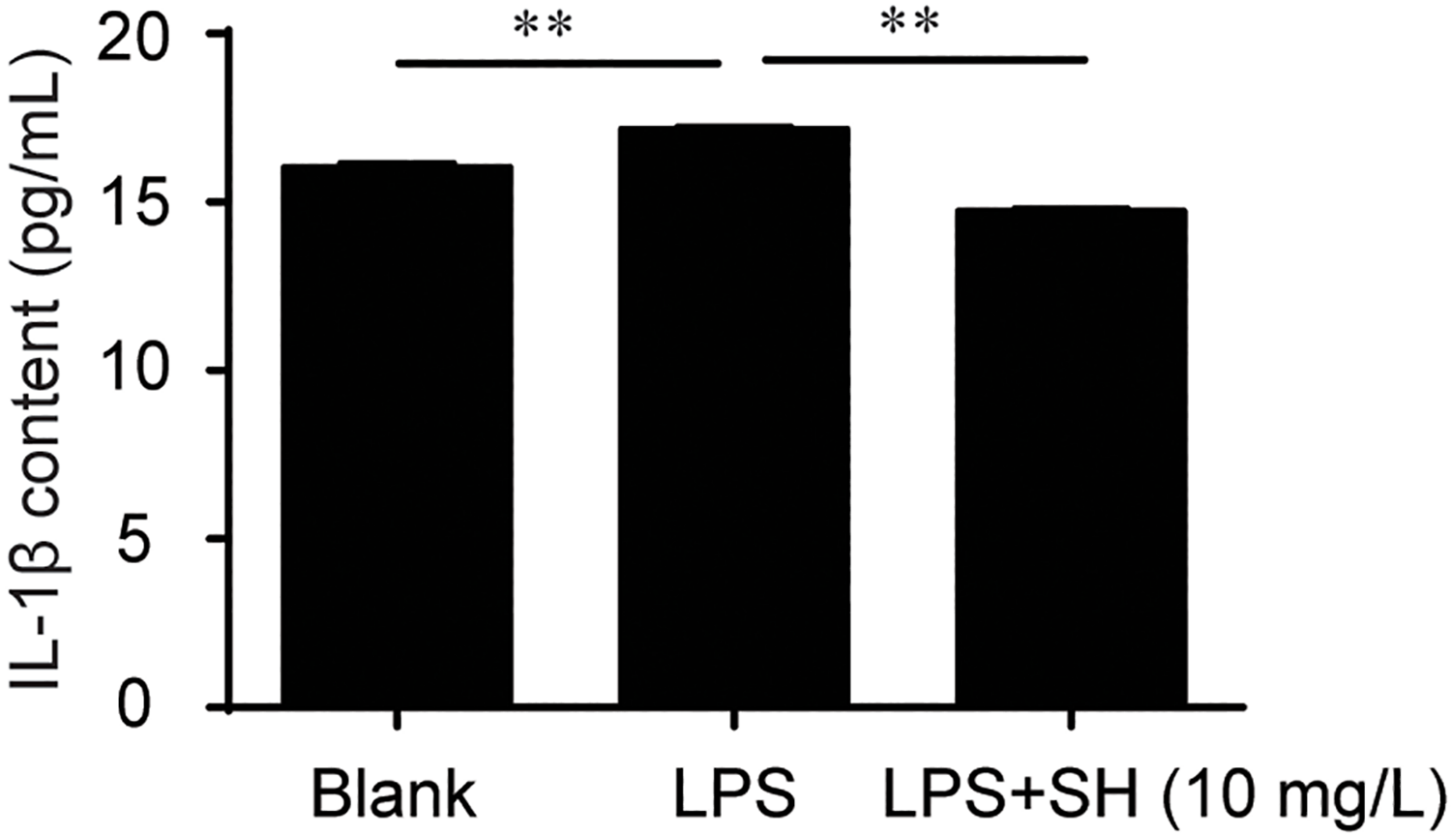
SH inhibited the LPS-elicited IL-1β production in RWA264.7 macrophages. Data are shown as means ± sem. **P<0.01.

We further investigated the direct inhibiting effects of SH on inflammatory factors production in RAW264.7 macrophages. The cells were pretreated with SH (10 mg/L) or solvent for 24 hours, then elicited by LPS for 4 hours. The production of IL-1β was quantified by ELISA kit. The results showed that treatment with SH (10 mg/L) significantly reduced the production of IL-1β elicited by LPS (Fig 4), suggesting that SH may inhibit inflammatory reaction of macrophages cells directly.

### SH decreased the phosphorylation of IkBα

**Fig 5:**
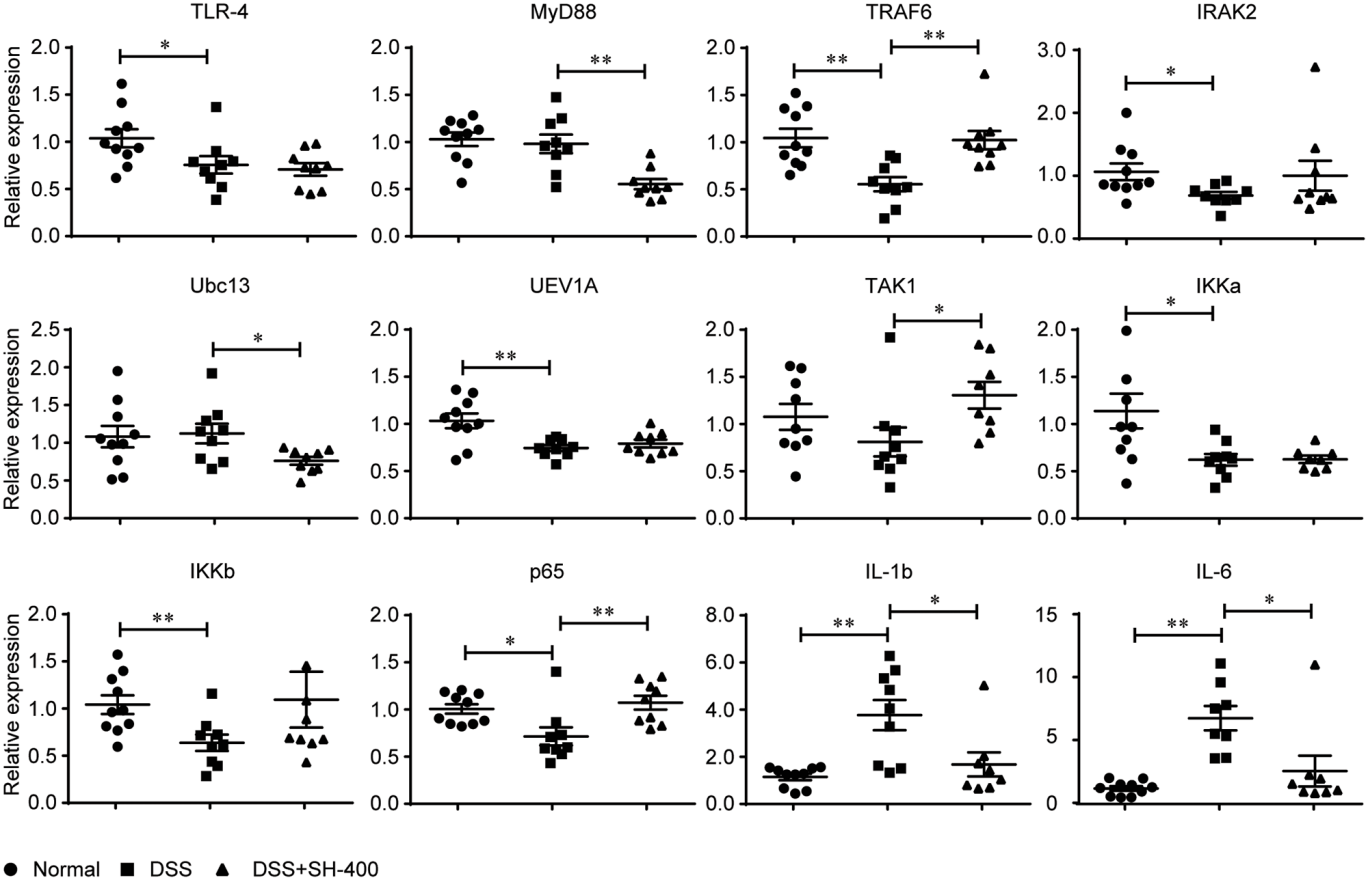
SH did not inhibit the transcription of TLR4-TRAF6-NFκB pathway. Data are shown as means ± sem. *P<0.05, **P<0.01.

To explore the potential mechanism underlying SH-mediated anti-inflammatory effects in DSS-induced colon colitis, we first quantified the mRNA levels of inflammatory genes in the TLR4-TRAF6-NFκB pathway by real-time PCR, that comprised, from the upstream to the downstream, TLR-4, MyD88, TRAF6, IRAK2, Ubc13, UEV1A, TAK1, IKKα/β and p65 (a subunit of NFκB). As shown in Fig 5, the transcription of most members except Ubc13 in the TLR4-TRAF6-NFκB pathway was not largely decreased by SH. However, the mRNA levels of IL-1β and IL-6 were significantly declined after SH treatment. These results indicated that the anti-inflammatory effects of SH were not due to the inhibition of transcription of TLR4-TRAF6-NFκB pathway.

IkBα is a key inhibitor of NFκB, which inhibits NFκB to enter the nucleus and initiate the expression of downstream inflammation-related genes such as IL-1β, IL-6, TNFα [20]. The action of IkBα is regulated by phosphorylation. The phosphorylated IkBα tends to be ubiquitinated and degraded, thus relieving the inhibition normally imposed on NFκB. We therefore evaluated the influence of SH on the phosphorylation levels of IkBα using western blot. The results showed that SH significantly decreased the phosphorylation of IkBα (Fig 3E), suggesting that SH may exert its anti-inflammatory effect by inhibiting IkBα phosphorylation, thus suppressing the activity of NFκB and decreasing inflammatory factors production.

### SH decreased the transcription of ASC3 and caspase-1

**Fig 6:**
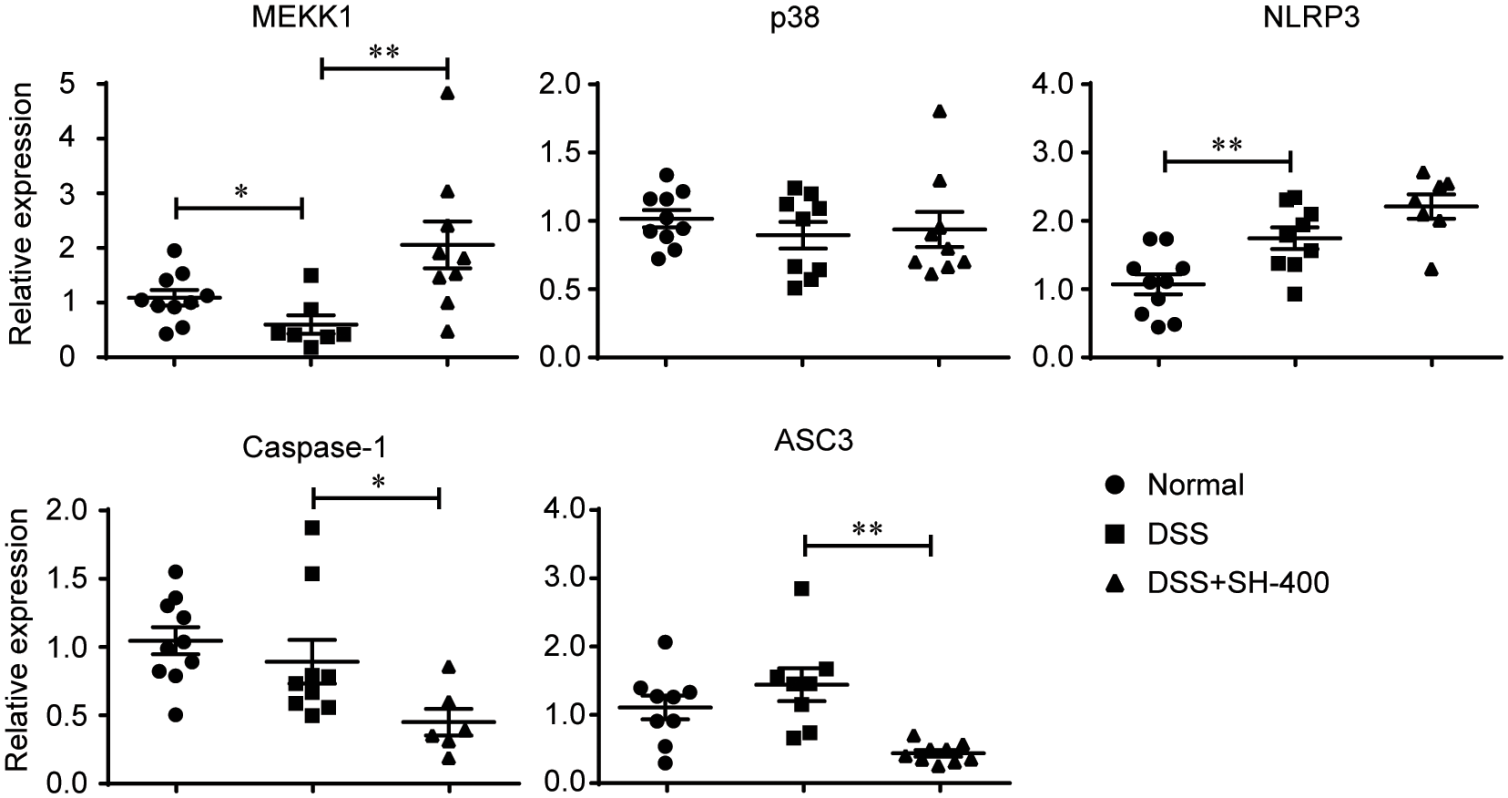
Effects of SH on the mRNA levels of genes in NLRP3-ASC3-caspase-1 pathway. Data are shown as means ± sem. *P<0.05, **P<0.01.

The NLRP3-ASC3-caspase-1 pathway is another inflammatory pathway which promotes the maturation and release of IL-1β and IL-6 cytokines [6]. We quantified the mRNA levels of NLRP3-ASC3-caspase-1 inflammatory pathway, which included MEKK, p38, NLRP3, ASC3 and caspase-1. The results showed that SH did not impact the transcription of p38 and NLRP3, increased the expression of MEKK1 and significantly decreased the mRNA levels of ASC3 and caspase-1 in DSS-induced colitis mice (Fig 6), suggesting that SH may inhibit release of inflammatory cytokines through downregulating ASC3-caspase-1 pathway.

## Discussion

Although multiple pharmacological activities of *P*. *igniarius* extract have been well-documented [7–14] but its effect on colitis is largely unknown. Recently, Song *et al* [21] reported that the ethanol and ethyl acetate extract of *P*. *linteus* exerted adequate colitis-ameliorating effect in DSS-induced colitis mice [21]. However, *P*. *igniarius* is traditionally used as decoction not by ethanol extraction. Therefore, whether the traditional usage of *P*. *igniarius* is beneficial for the treatment of colitis is remained to be elucidated.

Our results demonstrated that oral administration of aqueous extract of the fruiting body of *P*. *igniarius* (SH) is effective to ameliorate DSS-induced colitis in mice. Treatment with SH for 48 days significantly improved all clinical symptoms of DSS-induced chronic colitis including bodyweight reduction, DAI score, colon length and pathological changes such as abnormal crypts, loss of epithelial cells and goblet cells, and inflammatory infiltration. The dosage of *P*. *igniarius* aqueous extract (SH) we used in this study was similar to that used by Song *et al* for *P*. *linteus* ethanol and ethyl acetate extract (PBR). The effectiveness of SH on clinical symptoms of DSS-induced chronic colitis (48 days) was comparable to that of PBR on acute colitis (10 days). These results suggest that the decoction of *P*. *igniarius* is adequate to ameliorate DSS-induced colitis and thus provide a potential utility of Sanghuang for the treatment of colitis.

Colitis is a chronic remittent and relapsing inflammatory disease and multiple anti-colitis drugs exert their therapeutical effects through alleviating inflammation. Many *Phellinus* species have been demonstrated to possess potent anti-inflammatory activities [21–24] but whether *P*. *igniarius* (SH) possesses anti-inflammatory activity is still largely unknown. In this study, we showed that treatment with SH significantly and dose-dependently decreased the plasma levels of IL-6, IL-1β and TNF-a, suggesting that SH may ameliorate colitis through alleviating inflammation. Cell experiment showed that treatment with SH efficiently inhibited the LPS-elicited IL-1β production by RAW264.7 macrophages. These results suggested that SH could inhibit inflammation *in vitro* and *in vivo*.

Previous studies showed that some *Phellinus* species such as *P*. *linteus* [21, 25, 26] and *P*. *baumii* [23] exert their anti-inflammatory effects through inhibiting the expression of nuclear factor-κB (NFκB) p65 and mitogen-activated protein kinases (MAPKs) (e.g., extracellular signal-regulated protein kinase (ERK) and p38). We therefore quantified the transcription levels of all genes in LPS-elicited TLR4-TRAF6-NFκB inflammatory pathway in colon tissue. Our results showed that although the expression of IL-1β and IL-6 was significantly and dose-dependently by SH, but the mRNA levels of most genes in the TLR4-TRAF6-NFκB pathway such as TLR-4, TRAF6, IRAK2, UEV1A, TAK1, IKKα/β and p65 were not significantly decreased. We then checked the influence of SH on the phosphorylation of IkBα, a key inhibitor of NFκB which inhibits NFκB to enter the nucleus thus preventing the initiation of the expression of downstream inflammation-related genes [20]. Western blot showed that SH significantly decreased phosphorylation level of IkBα. As the phosphorylated IkBα is prone to degraded, inhibiting IkBα phosphorylation would enhance the inhibitory action of IkBα on NFκB, thus decreasing the expression of downstream inflammatory genes such as IL-1β and IL-6.

The NLRP3-ASC3-caspase-1 pathway is another important inflammatory pathway that promotes the production and release of IL-1β and IL-6 pro-inflammatory cytokines [6]. Treatment with SH did not decrease the transcription of MEKK, p38 and NLRP3, but significantly decreased the mRNA levels of ASC3 and caspase-1. As ASC3 and caspase-1 are key regulators for the maturation of IL-1β and IL-6 [6], SH may reduce the production and release of these two pro-inflammatory cytokines through inhibiting ASC3 and caspase-1. Therefore, the aqueous extract of *Phellinus igniarius* (SH) may suppressing the plasma levels of IL-1β and IL-6 through two pathways, (1) decreasing the phosphorylation of IkBα thus preventing NFκB to initiate the expression of downstream inflammatory genes, (2) inhibiting the expression of ASC3 and caspase-1 to reduce the maturation of IL-1β and IL-6, which ultimately ameliorates DSS-induced colitis in mice.

In conclusion, our work demonstrated that the aqueous extract of *Phellinus igniarius* (SH) effectively ameliorates DSS-induced colitis, which provides a potential utility of SH for the treatment of colitis. Inhibiting the expression and release of inflammatory factors may participate in the colitis-ameliorating effect of SH.
